# Single-Beat Noninvasive Imaging of Ventricular Endocardial and Epicardial Activation in Patients Undergoing CRT

**DOI:** 10.1371/journal.pone.0016255

**Published:** 2011-01-27

**Authors:** Thomas Berger, Bernhard Pfeifer, Friedrich F. Hanser, Florian Hintringer, Gerald Fischer, Michael Netzer, Thomas Trieb, Markus Stuehlinger, Wolfgang Dichtl, Christian Baumgartner, Otmar Pachinger, Michael Seger

**Affiliations:** 1 Division of Cardiology, Department of Internal Medicine III, Medical University Innsbruck, Innsbruck, Austria; 2 Institute of Electrical, Electronic and Bioengineering, University for Health Sciences, Medical Informatics and Technology (UMIT), Hall in Tirol, Austria; 3 Department of Radiology I, Medical University Innsbruck, Innsbruck, Austria; Cornell University, United States of America

## Abstract

**Background:**

Little is known about the effect of cardiac resynchronization therapy (CRT) on endo- and epicardial ventricular activation. Noninvasive imaging of cardiac electrophysiology (NICE) is a novel imaging tool for visualization of both epi- and endocardial ventricular electrical activation.

**Methodology/Principal Findings:**

NICE was performed in ten patients with congestive heart failure (CHF) undergoing CRT and in ten patients without structural heart disease (control group). NICE is a fusion of data from high-resolution ECG mapping with a model of the patient's individual cardiothoracic anatomy created from magnetic resonance imaging. Beat-to-beat endocardial and epicardial ventricular activation sequences were computed during native rhythm as well as during ventricular pacing using a bidomain theory-based heart model to solve the related inverse problem. During right ventricular (RV) pacing control patients showed a deterioration of the ventricular activation sequence similar to the intrinsic activation pattern of CHF patients. Left ventricular propagation velocities were significantly decreased in CHF patients as compared to the control group (1.6±0.4 versus 2.1±0.5 m/sec; p<0.05). CHF patients showed right-to-left septal activation with the latest activation epicardially in the lateral wall of the left ventricle. Biventricular pacing resulted in a resynchronization of the ventricular activation sequence and in a marked decrease of total LV activation duration as compared to intrinsic conduction and RV pacing (129±16 versus 157±28 and 173±25 ms; both p<0.05).

**Conclusions/Significance:**

Endocardial and epicardial ventricular activation can be visualized noninvasively by NICE. Identification of individual ventricular activation properties may help identify responders to CRT and to further improve response to CRT by facilitating a patient-specific lead placement and device programming.

## Introduction

Cardiac resynchronization therapy (CRT) has evolved as an established treatment in patients with severe heart failure refractory to optimized neurohumoral therapy. Large clinical trials showed a significant benefit on mortality and on morbidity in patients with wide QRS complex in NYHA class III and severely impaired left ventricular ejection fraction (LVEF) [1].

There are many studies about electrophysiologic and mechanical mechanisms of CRT. Despite the fact that during CRT the right ventricular lead is placed endocardially and left ventricular pacing is performed using an epicardial lead placed within the coronary veins, little is known about endocardial and epicardial activation in these patients. Invasive catheter based mapping of ventricular activation reflects only right- and left ventricular endocardial sites. Information on epicardial activation is limited to a small area of the left ventricle accessible via mapping in the coronary sinus. Most of these data were obtained by conventional fluoroscopy guided electrophysiological mapping as well as by electromagnetic three-dimensional non-fluoroscopic electroanatomic contact mapping [2], [3].

This study was conducted to characterize biventricular endocardial and epicardial activation in heart failure patients undergoing CRT compared to a healthy control group using a novel noninvasive cardiac mapping tool –Noninvasive Imaging of Cardiac Electrophysiology (NICE).

## Methods

### Magnetic resonance imaging

Prior to CRT-device implantation or electrophysiologic study, patient-specific anatomic data were obtained by magnetic resonance imaging (MRI) using a Magnetom Vision Plus 1.5 Tesla scanner (Siemens, Erlangen, Germany). MRI was performed during the morning clinical routine, whereas the procedures respectively the measurements were performed in the afternoon with a mean delay of about 2.5 hours. Ventricular end-diastolic geometry and torso geometry were assessed in ECG-gated cine mode during breath-hold as described previously [4], [5]. Liquid-filled anatomic markers (vitamin E capsules) on the patient's torso were used to couple the MRI geometric data with the data obtained during electrophysiologic examination.

### ECG mapping

A high-resolution 65-lead electrode array was applied in the catheter laboratory before the ablation procedure. Radiotranslucent carbon electrodes were used to facilitate simultaneous fluoroscopy. ECG recordings were performed using the Mark-8 recording system (Biosemi V.O.F., Amsterdam, Netherlands) at a sampling rate of 2048 Hz (0.3 Hz to 400 Hz band pass filter) and an AC resolution of 500 nV/bit (16 bit AD converter, i.e. 32 mV AC input range). The Mark-8 recording system is a battery-powered (6 Volts) high-precision ECG amplifier. It was fixed at bedside to keep wire connections as short as possible. Data were transferred to the recording PC via an optic fiber.

### Anatomic coupling

As anatomical data were recorded in the MRI frame, all other geometric data also had to be transformed in this frame. For this purpose seven anterior and lateral MRI markers and the anterior and lateral electrode positions were measured with a magnetic digitizer (Fastrak^TM^, Polhemus Inc., Colchester, VT, USA). The electrode positions were transformed by a rigid body transformation (rotation matrix and displacement vector) minimizing the root-mean-square (RMS) distance of the seven Polhemus marker positions to the MRI marker positions. The posterior electrodes were placed at the location of the posterior markers.

### Noninvasive imaging of cardiac electrophysiology (NICE)

**Figure 1:**
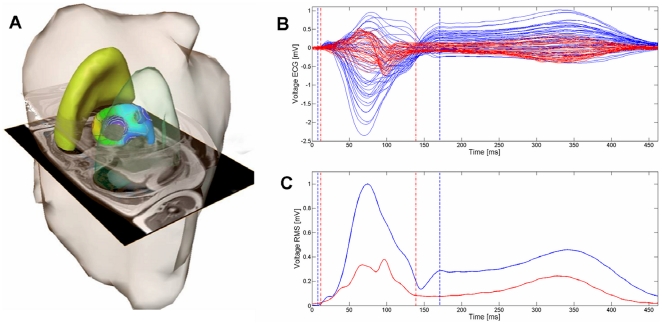
Panel A shows a patient-specific anatomical model obtained from MRI data (two MRI image slides shown as intersection planes) including chest, lungs and the ventricles merged with the inverse calculated electroanatomic activation map (isochrones; color coded). Panel B shows a butterfly plot of the 65-body surface ECG leads during native sinus rhythm (blue lines) and biventricular pacing (red lines). Panel C shows a root mean square (RMS) plot (sinus rhythm – blue line; biventricular pacing – red line; dotted lines indicate begin/end of the QRS complex).

NICE requires assessment of the patient's anatomy (MRI) in order to construct a patient-specific computer model and a high-resolution ECG map providing functional information on target beats. For each patient an individual computer model including compartments of different conductivity (heart, lung, blood mass and chest surface) was constructed. For this purpose, a commercial software package (AMIRA Developer, TGS Template Graphics Software, Inc., Merignac, France) was adapted for contour detection and segmentation as previously described (**Figure 1**, **Movie S1**) [5], [6].

A bidomain model based boundary element formulation (linear triangular elements) was applied to relate step-like local activation (resting potential: −90 mV; plateau level: 0 mV; rising time: 3 ms) at the endocardial and epicardial source points to the simulated potentials at the electrode locations [7]. A Wilson terminal defined the reference for measured and computed unipolar signals [8].

For inverse computation of the ventricular activation sequence the target beats were selected using an automated signal processing algorithm as previously described [9]. In brief, the model-based computation of the activation sequence of a single beat can be described as follows: For an assumed model activation sequence the ECG is simulated and compared with the measured ECG. The activation sequence is then systematically changed to minimize the difference between the simulated and the measured data. The optimization routines described in [4], [7] were used to compute the activation sequence that best fits the measured data. The initial estimation of the activation sequence was computed applying the critical point theorem [10]. As this imaging problem was ill-posed (i.e., the solution was sensitive to noise and model error), an additional regularization term of the Tikhonov second-order type was considered in the optimization process. This regularization imposes the constraint that neighboring source points have similar activation times (smooth activation pattern). The coupled regularization strategy was applied to solve the nonlinear optimization problem [4], [7]. Details on the NICE method have been published previously [4], [5], [7], [11].

### Data acquisition

In the CHF patients NICE was performed during native sinus rhythm (no pacing), right ventricular (RV) and biventricular pacing (biV). In the control patients NICE was performed during intrinsic rhythm as well as during RV pacing via a quadripolar mapping catheter in an apical position. In all patients right and left ventricular total activation duration, earliest septal, endocardial and epicardial breakthroughs, endocardial/epicardial activation sequences were analyzed during native rhythm as well as during the different pacing modes. Atrial synchronous pacing was perfomed in all patients and the AV-interval was optimized either by impedance cardiography or echocardiographically according to Ritter et al [12]. The breakthrough sites were identified as the sites on the ventricular epicardium/endocardium where depolarization first appeared. Ventricular total activation duration was defined as the interval from the earliest breakthrough to the latest observed electrical activation of the right and left ventricle. Root-mean-square QRS duration was measured during native sinus rhythm as well as during different pacing modes as previously described [9].

### Statistical analysis

Results are shown as mean ± SD and are expressed as absolute values. Statistical analysis of the data was performed with SPSS 10.1 for Windows (SPSS Inc., Chicago, Illinois). A two-sided Students *t*-test (unpaired for independent samples, paired for dependent samples) was used to evaluate the results, and a p value <0.05 was considered statistically significant.

The study was approved by the ethics committee of the Medical University Innsbruck (UN3742 281/4.2), and written, informed consent was obtained from all patients.

## Results

### Patient population

Ten patients (1 female, mean age 63±6 years, NYHA class III, LVEF <35%) with congestive heart failure (ischemic n = 2) and left bundle branch block (LBBB) undergoing CRT and ten patients (4 females; mean age 31±16 years, LVEF >50%) without structural heart disease and normal atrioventricular conduction undergoing an EP study (control) were studied (**Table 1**). At the time of evaluation, all CHF patients were clinically stable without signs of fluid overload and each patient was on optimized neurohumoral therapy. None of the control subjects received regular medication. All CHF patients received ACE inhibitor/AT blocker (100%), 9/10 of the patients were on betablocker therapy and took diuretics on a regular basis (90%).

**Table 1:** MRI characteristics of control versus LBBB patients.

	RVEF [%]	LVEF [%]
control	51±9	62±10*
LBBB	38±12	22±9*

(RVEF, right ventricular ejection fraction; LVEF, left ventricular ejection fractionion; * indicate a p-value <0.05).

### Endocardial and epicardial ventricular activation in healthy subjects

**Figure 2:**
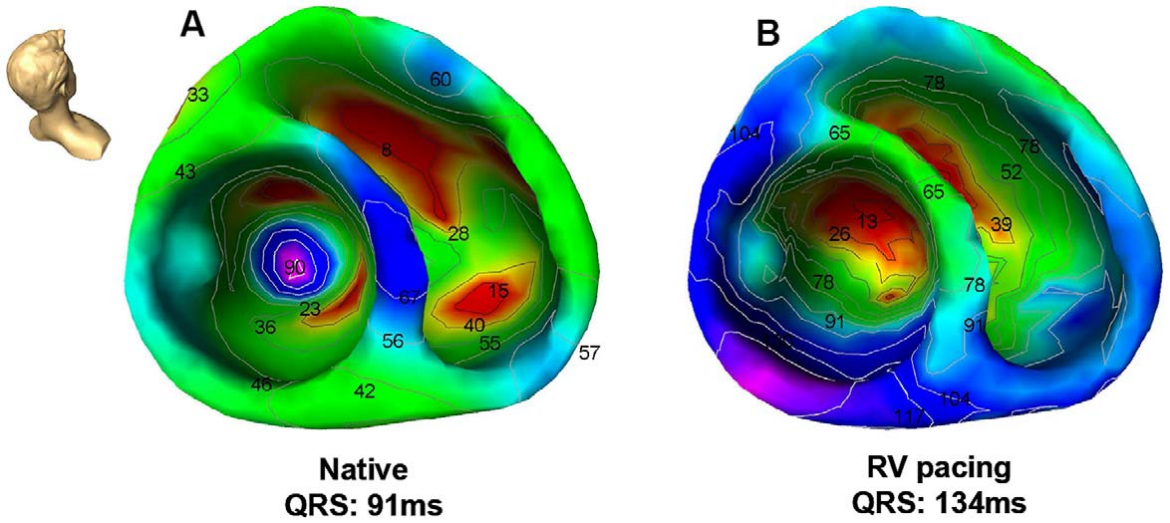
Color-coded electroanatomic activation map of the right and left ventricle in a control patient (no structural heart disease) during A) intrinsic sinus rhythm (native) and B) right ventricular (RV) pacing. Red color illustrates earliest activation, blue color illustrates area of late ventricular activation. Head icon indicates point of view.

During native sinus rhythm earliest ventricular activation was observed endocardially in the right ventricular free wall with the earliest septal breakthrough site located in the mid-septal area of the left ventricle in 8 out of 10 patients. In 2 patients the site of the earliest septal breakthrough was located in an apical-septal position. No patient showed a septal breakthrough at a basal-septal position in the left ventricle. Left septal activation was followed by left ventricular endocardial and right ventricular mid-septal activation. Epicardial activation was observed in both ventricles immediately after endocardial activation (**Table 2** **,** **Figure 2 A**, **Movie S2**, **Movie S3**).

**Table 2:** Endocardial and epicardial left- and right-ventricular breakthroughs during different pacing modes.

	RV endo [ms]	RV epi [ms]	LV endo [ms]	LV epi [ms]	RV septal [ms]	LV septal [ms]
control native	12±13‡	19±13‡	17±10#‡	24±16#‡	20±10‡	16±10#‡
control RV	0±1‡	14±6‡	41±13‡	36±16‡	12±11‡	34±11‡
LBBB native	7±10§	10±8†	46±19#†	49±16#†	17±12†	36±13#
LBBB RV	0±0*§	15±6*	50±18*	51±17*	16±6*	37±10
LBBB biV	16±13*	28±12*†	17±7*†	1±2*†	30±11*†	40±11

(Native, no stimulation; RV, right ventricular pacing; biV, biventricular pacing; *§*,*,†,‡,# indicate a p-value <0.05).

During RV pacing the ventricular activation pattern changed significantly. The activation of the left ventricle became more delayed as compared to native sinus rhythm (endocardial: 17±10 versus 41±13 ms, epicardial: 24±16 versus 36±16 ms; both p<0.05). During RV pacing right ventricular septal activation was preceded (20±10 versus 12±11 ms; p<0.05) and the breakthrough site shifted from a mid-septal to an apical-septal position. Also the septal breakthrough site of the left ventricle was markedly delayed (16±10 versus 34±11 ms; p<0.05) as compared to native rhythm. In 2 patients the LV septal breakthrough sites changed form mid-septal during intrinsic conduction to an apical-septal site during RV pacing (**Table 2** **,** **Figure 2B**, **Movie S4**, **Movie S5**).

### Endocardial and epicardial ventricular activation in heart failure patients

**Figure 3:**
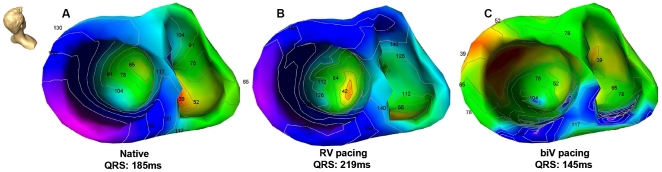
Color-coded electroanatomic activation map of the right and left ventricle in a CHF patient during A) intrinsic sinus rhythm (native), B) right ventricular (RV) and C) biventricular (biV) pacing. Red color illustrates earliest activation, blue color illustrates area of late ventricular activation. Head icon indicates point of view.

Similar to the control group also in the CHF patients the earliest ventricular activation was located in the right ventricular endocardium. The earliest septal breakthrough site was located in an apical position of the right septum in 5 patients. In the remaining patients right septal breakthrough sites were located in the mid-septum. The right septum was activated from apical/mid-septal to basal. Due to complete LBBB left ventricular endocardial and epicardial activation was markedly delayed in each patient as compared to control patients (endocardial: 46±19 versus 17±10 ms; epicardial: 49±16 versus 10±8 ms; both p<0.01). Left septal endocardial breakthroughs were located in a mid-septal position. One patient showed the earliest LV septal breakthrough in an apical position and in another patient the breakthrough site was located in a basal position. In all CHF patients the left ventricular activation wavefront showed a similar pattern. It turned around the apex and spread radially across the inferior/anterior wall towards the lateral wall of the left ventricle. Although the site of latest ventricular activation was located epicardially in the lateral wall of the left ventricle in all patients, individual differences in the orientation of the area of latest activation (from anterolateral to posterolateral) were obvious (**Table 2** **,** **Figure 3A**, **Movie S6**, **Movie S7**). Right ventricular pacing showed no significant effects on septal and LV activation as compared to native rhythm. It resulted in a decrease of the time course of RV endocardial breakthrough, a reversal of RV endocardial and epicardial activation and a marked prolongation of the total activation duration of both the left and right ventricles. The effect of RV pacing on the activation of the left ventricular lateral wall was inhomogeneous in CHF patients. Some patients showed no changes whereas in others the spreading of the wavefront and the sites of latest activation shifted from (antero)lateral to (postero)lateral as compared to native sinus rhythm (**Table 2** **,** **Figure 3B**, **Movie S8**, **Movie S9**). Biventricular pacing showed a relative prolongation of RV epicardial activation (28±12 versus 10±8 ms; p<0.05) and a prolongation of the RV septal activation times (30±11 versus 17±12 ms; p<0.05) as compared to native rhythm. During biventricular pacing the onset of LV endocardial as well as epicardial activation was markedly shortened as compared to native sinus rhythm (17±7 and 1±2 ms versus 46±19 and 49±16 ms; both p<0.01). During biventricular pacing the ventricular activation sequence markedly changed. The earliest activation was located epicardially in the LV lateral free wall. This was followed by an endocardial breakthrough located in a RV apical septal position. The LV septal breakthrough site shifted from mid-septal to apical-septal in most of the patients. Therefore, biventricular activation accelerated and resulted in an improvement of ventricular synchrony (**Table 2** **,** **Figure 3C**, **Movie S10**, **Movie S11**). Biventricular depolarization as indicated by RMS QRS duration was shortened during biventricular pacing as compared to native sinusrhythm and RV pacing (134±26 versus 155±28 and 179±31 ms; p<0.05). Moreover, total activation duration of the left ventricle was significantly decreased during biventricular pacing as compared to RV pacing and native sinus rhythm (129±16 versus 173±25 and 157±28 ms; both p<0.05) (**Table 3**).

**Table 3:** Left- and right ventricular total activation duration during native rhythm and during right- and biventricular pacing.

	TAD LV [ms]	TAD RV [ms]
control native	95±14*§*‡	89±15*
control RV	125±11‡	122±14*
LBBB native	157±28*§*†	102±19
LBBB RV	173±25	128±48
LBBB biV	129±16†	108±23

(TAD LV, left ventricular total activation duration; TAD RV, right ventricular total activation duration; native, no stimulation; RV, right ventricular pacing; biV, biventricular pacing; *§*,*,†,‡ indicate a p-value <0.05).

### Differences in native ventricular activation between control and heart failure patients

During native sinus rhythm there were no differences in the timing of right ventricular septal breakthroughs between control and LBBB patients (20±10 versus 17±12 ms; p = ns) whereas left ventricular septal breakthroughs were delayed in LBBB patients (16±10 versus 36±13 ms; p<0.05). The site of the latest right ventricular activation was located in both groups in the majority of patients in the basal portion of the right ventricle. Moreover, during native sinus rhythm the septum was activated from left to right in control patients whereas this sequence was reversed in CHF patients (similar septal activation pattern as during RV pacing in control patients). In both groups left ventricular endocardial activation started in the septum. Due to LBBB RMS QRS duration was increased in CHF patients as compared to control patients (155±29 versus 101±12 ms; p<0.01). Also LV total activation duration was increased as compared to control patients (157±28 versus 95±14 ms, p<0.01). There was no significant difference in right ventricular total activation duration between CHF and control patients (**Table 3**). Left ventricular endocardial and epicardial breakthroughs were significantly delayed in CHF patients as compared to control patients (46±19 and 49±16 ms versus 17±10 and 24±16 ms; both p<0.01) (**Table 2**). Left ventricular propagation velocities were significantly decreased in the CHF patients as compared to control patients (1.6±0.4 versus 2.1±0.5 m/sec; p<0.05). There was no difference in intrinsic propagation velocities of the right ventricle between both groups (1.7±0.2 versus 1.9±0.4 m/sec; p = ns) (**Table 1**).

## Discussion

This is the first study which simultaneously evaluated endocardial as well as epicardial (bi)ventricular activation during native sinus rhythm as well as during different ventricular pacing modes by using a novel noninvasive electroanatomical imaging method (NICE). Due to limitations of current mapping techniques, there are only limited data available about the effects of different pacing modes on biventricular endocardial and epicardial activation in humans [2], [13]. As previously described, NICE facilitates individual beat-to-beat electroanatomic mapping by inverse computation of the ventricular activation sequence applying a mathematical algorithm and fusion of the data with an individual heart model to solve the underlying ill-posed problem [14]. Up to date, considerable efforts have been undertaken to solve the inverse problem of electrocardiography to localize and image cardiac activation sequences from body surface electrocardiograms [15], [16], [17]. Previous studies have shown that the electrical excitation of the heart can be reconstructed by fusing data from surface ECG mapping and MRI [5]. Nevertheless, most of these studies are either limited with respect to clinical applicability (conducted in animals) or deliver an incomplete depiction of cardiac electrical activation (e.g. visualization of epi- or pericardial activation only).

In our study control patients showed a deterioration of the ventricular activation sequence during RV pacing comparable to the activation sequence of CHF patients with complete LBBB during native sinus rhythm. During RV pacing the septal, as well as the endocardial and epicardial activation times of the left ventricle were markedly delayed as compared to native sinus rhythm. The septal activation sequence changed from left-to-right during native conduction to a right-to-left septal activation pattern which is in accordance to previous data [18]. The earliest LV activation was observed close to the septum reproducing the activation pattern of LBBB patients. Moreover, RV pacing resulted in an increase of total RV and LV activation duration. This increase in ventricular activation duration during RV pacing may be due to cell-to-cell coupled propagation of the activation wavefront until connecting to the intrinsic conduction system and spreading via the Purkinje system.

In CHF patients the delay between right and left ventricular endocardial and epicardial activation was significantly prolonged due to complete LBBB as compared to control patients. In CHF patients biventricular pacing did not affect the direction of transseptal activation respectively the timing of the LV septal breakthrough although the RV septal breakthrough was delayed as compared to native sinus rhythm. This indicates that the major effect of CRT is achieved by preceding the left ventricle by stimulation close to the site of latest left ventricular activation rather than affecting propagation via the intrinsic conduction system. Therefore, optimal placement of the left ventricular lead is crucial and stimulation as close as possible to the site of latest left ventricular activation (during native rhythm) should be aimed at. Epicardial pacing at the left lateral wall and simultaneous endocardial pacing at the right ventricular apex during CRT resulted in a significant decrease of LV total activation duration. Interestingely, total activation duration of the right ventricle was not changed during CRT. This study revealed also differences in propagation velocities of the ventricular activation between the CHF and control group. Left ventricular propagation velocities were significantly decreased in CHF patients as compared to control patients. This is in accordance with the results by Rodriguez et al. [18] who found reduced conduction velocities in patients with congestive heart failure. Both CHF and control patients did not show a significant difference in propagation velocities of the right ventricles. This finding may indicate that the underlying substrate of ventricular dyssynchrony was located mainly within the left ventricle in the CHF patients included into our study. This may be different in patients with arrhythmogenic heart disease or hypertrophic cardiomyopathy.

Despite the increase of both right and left ventricular total activation duration during RV pacing in control as well as in CHF patients, the absolute increase of left ventricular total activation duration was much more pronounced in CHF patients. In addition, the transseptal activation times were significantly prolonged in CHF patients as compared to control patients. This may cause additional dyssynchrony of papillary muscle contraction and thus, increase the mitral regurgitation in these already compromised patients. In accordance to a previous study by Aurricchio et al. [2] our data showed a specific pattern of the left ventricular activation sequence in CHF patients with LBBB. In all CHF patients with LBBB the activation wavefront turned around the apex and inferior wall of the left ventricle. The sites of the latest left ventricular activation were inhomogenous within the LBBB patients. Latest ventricular activation was found epicardially in the LV lateral wall with individual differences in the orientation of the area of latest activation. This inhomogeneity may reflect the individual differences in the underlying substrate of heart failure. This typical ventricular activation pattern in LBBB patients may be due to a slowing of conduction within the intrinsic conduction system, a prolongation of intramural activation times because of areas of slow conduction tissue or altered cell-to-cell coupling, which therefore results in a kind of functional block [19]. Interestingly, this activation pattern was also visible in control patients during RV pacing. This indicates that also RV pacing in patients without structural heart disease results in similar activation patterns comparable to CHF patients with LBBB. This kind of functional block in the LV is dependent on pacing site and can be reversed by biventricular stimulation. Due to impaired conduction abilities and cell-to-cell coupling properties unphysiologic RV pacing show more detrimental effects in CHF patients as compared to RV pacing in patients without structural heart disease. Patients without structural heart disease may be able to temporarily compensate the negative effects of RV pacing on septal and left ventricular depolarization whereas in CHF patients the functional reserve is limited and the negative effects on hemodynamic behavior are immediately obvious [20].

However, in CHF patients deterioration of ventricular activation due to complete LBBB can be reversed by stimulation close to the site of latest left ventricular activation. Most of the time this can be achieved using an epicardial lead which is positioned in a posterolateral branch of the coronary veins. NICE facilitates the identification of target sites for optimal lead placement and may also be helpful in determining responders to CRT. Current guidelines for selection of patients for CRT are based on QRS duration and NICE may have the potential to help to refine this process as it enables visualization of ventricular electroanatomic activation noninvasively. Nevertheless, simultaneous endocardial RV and epicardial LV pacing during CRT results in a different activation pattern as compared to native activation via the intrinsic conduction system. There are some controversial data about potential proarhythmogenic effects of CRT due to the reversal of epicardial and endocardial activation [9], [21]. However, prospective randomized studies did not find any excess mortality due to sudden death during biventricular pacing [22]. Nevertheless, patients with reduced ventricular function have an increased risk of malignant ventricular tachyarrhythmias. Catheter ablation of the substrate of such a tachycardia is feasible but the procedure can be complicated if the focus of the arrhythmia is located within the epicardium. NICE has the potential to discriminate whether it is of epicardial or endocardial origin and it may therefore be useful for planning such procedures. Previous data have shown that biventricular pacing reduces mitral regurgitation [23], although the mechanism of CRT on mitral regurgitation is still not well understood. A study by Agricola et al [24] suggests that mitral regurgitation is related to the presence of LV dyssynchrony that involves the posterior mitral leaflet. As shown in this study the transseptal activation in CHF patients with LBBB is not reversed by CRT but the LV septal breakthrough site changed from mid-septal to apical-septal. This shift in LV septal breakthrough site may result in a resynchronization of the posterior mitral valve leaflet (via activation of the medial papillary muscle) and therefore reduce mitral regurgitation. Nevertheless, for a better understanding this should be addressed by future studies. One limitation of this study is that delayed enhancement imaging was not performed and the relationship between scar characteristics and activation properties were not look at. Delayed enhancement imaging has been shown to predict the response to cardiac resynchronization therapy in patients with intraventricular dyssynchrony. Therefore, the relationship between scar characteristics and activation properties warrants further investigation.

Noninvasive imaging of (bi)ventricular endocardial and epicardial acivation is feasible by using NICE. NICE is effective in characterization of individual LV activation properties and may therefore help to further improve the identification of responders to CRT. Moreover, NICE has the potential to improve the response to CRT by facilitating an individual patient-specific pacemaker therapy.

## Supporting Information

Movie S1 Movie clip shows the NICE workflow in a healthy subject. A patient-specific heart volume conductor model of the thorax is build by MRI data. Data derived from high-resolution body surface ECG allows inverse calculation of electroanatomic activation of the heart. At the beginning of each file the red color illustrates earliest activation, blue color illustrates the area of late ventricular activation. Head icon indicates point of view.
(WMV)

Movie S2 NICE activation maps during native rhythm in a control patient with a view on the left septum.
(MPG)

Movie S3 NICE activation maps during native rhythm in a control patient with a view on the right septum.
(MPG)

Movie S4 NICE activation maps of both ventricles in a control patient during RV pacing with a view on the left septum. At the beginning of each file an isochronal activation map is shown where red color illustrates earliest activation, blue color illustrates areas of late ventricular activation. Head icon indicates point of view. “T  = ” shows the activation time in milliseconds.
(MPG)

Movie S5 NICE activation maps of both ventricles in a control patient during RV pacing with a view on the right septum. At the beginning of each file an isochronal activation map is shown where red color illustrates earliest activation, blue color illustrates areas of late ventricular activation. Head icon indicates point of view. “T  = ” shows the activation time in milliseconds.
(MPG)

Movie S6 NICE activation maps during native rhythm in a CHF patient with a view on the left septum.
(MPG)

Movie S7 NICE activation maps during native rhythm in a CHF patient with a view on the right septum.
(MPG)

Movie S8 NICE activation maps of both ventricles in a CHF patient during RV pacing with a view on the left septum.
(MPG)

Movie S9 NICE activation maps of both ventricles in a CHF patient during RV pacing with a view on the right septum.
(MPG)

Movie S10 NICE activation maps in a CHF patient during biV pacing with a view on the left septum. At the beginning of each file an isochronal activation map is shown where red color illustrates earliest activation, blue color illustrates areas of late ventricular activation. Head icon indicates point of view. “T  = ” shows the activation time in milliseconds.
(MPG)

Movie S11 NICE activation maps in a CHF patient during biV pacing with a view on the right septum. At the beginning of each file an isochronal activation map is shown where red color illustrates earliest activation, blue color illustrates areas of late ventricular activation. Head icon indicates point of view. “T  = ” shows the activation time in milliseconds.
(MPG)
